# Maxillary Expansion With C‐Expander Using Digital Planning and CBCT‐Based Surgical Guidance: A Case Report

**DOI:** 10.1155/crid/3316626

**Published:** 2026-10-06

**Authors:** Enrique Miranda-Astocondor, Alberto Rafael Ríos-Quimper, Marjorie Regina Eguren-Langer

**Affiliations:** ^1^ Faculty of Dentistry National University Federico Villarreal Lima Peru; ^2^ Faculty of Dentistry University Norbert Wiener Lima Peru

**Keywords:** case reports, miniscrew-assisted maxillary expansion, orthodontic, orthodontic appliance, palatal expansion technique

## Abstract

This clinical case report describes the clinical management of a patient with a transverse maxillary deficiency treated with a C‐expander. The objective is to present the diagnosis, tomographic planning, implantation, and postexpansion evolution. The patient presented with typical characteristics of a transverse maxillary deficiency, with a maxillomandibular discrepancy of 6.8 mm; the insertion of miniscrews was planned using cone‐beam computed tomography (CBCT) and intraoral scanning with BlueSky Plan 4 software. A surgical guide was fabricated, and the C‐expander was installed; activation was performed progressively under weekly clinical monitoring. Another CBCT scan was performed after the expansion. Follow‐up CT showed transverse improvement with a nearly parallel opening pattern of the median palatine suture of approximately 6.5 mm on the axial view. Dentoalveolarly, the upper intermolar distance increased by 7.1 mm and the lower by 1 mm. Intermolar angulation increased by 2° in the upper arch and 5° in the lower arch; overjet increased by 1 mm and overbite decreased by 15%. Furthermore, the 3D frontal facial reconstruction revealed an increase in the intercanthal distance of 0.3 mm, an increase in the nasal interalar distance of 3.5 mm, and an increase in the nasal base width of 4.4 mm. In conclusion, maxillary expansion using a digitally guided C‐expander successfully achieved separation of the midpalatal suture in this patient, with limited dental and facial effects during the immediate postexpansion.

## 1. Introduction

Transverse maxillary deficiency is a frequent problem in orthodontic practice, occurring in 9%–23% of cases [1]. It is commonly associated with unilateral or bilateral posterior crossbite. Still, in cases of dental compensation, it manifests as a narrow smile or an accentuated curve of Wilson due to the buccal inclination of the upper posterior teeth and the lingual inclination of the lower posterior teeth [2, 3]. The treatment of choice in these cases is rapid maxillary expansion (RME), with over 150 years of reports in orthodontics [2, 4–6], which is aimed at expanding the maxilla by separating the midpalatal suture and the circummaxillary and circumzygomatic suture system [7, 8].

RME becomes increasingly challenging as sutural maturation occurs. Traditionally, RME provides dental support, which, with greater sutural closure, would lead to a lesser skeletal effect accompanied by undesirable side effects such as tooth tilting. Root resorption, marginal bone loss, and decreased vestibular bone thickness are common in these cases [9, 10]. Historically, the treatment alternative for adults in these cases was surgical‐assisted maxillary expansion (SARPE). However, with the advent of skeletal anchorage and cone‐beam computed tomography (CBCT) for more precise diagnosis and planning, it is now possible, in some situations, to break the median palatine suture without surgery [10–13].

To achieve effective nonsurgical expansion, miniscrew‐assisted rapid palatal expansion (MARPE) was introduced. Although traditional MARPE incorporates hybrid anchorage supported by both teeth and miniscrews [14, 15], its effectiveness may be limited in patients with thin parasutural bone or a vulnerable periodontal phenotype. To overcome these limitations, the C‐expander—a fully implant‐supported maxillary expander placed laterally in the palatal alveolar process—has been recently proposed. By eliminating tooth anchorage, it allows for greater flexibility in miniscrew placement; furthermore, it is compatible with any miniscrew system and is more cost‐effective than prefabricated MARPEs [16–20].

Despite favorable preliminary reports, clinical evidence describing CBCT‐based planning, insertion protocols, and three‐dimensional outcomes of the C‐expander remains limited, particularly in patients in whom conventional MARPE protocols are anatomically restricted by severe parasutural bone atrophy (< 1.5‐mm thickness) or nasal floor variations that preclude traditional paramedian placement. Addressing this critical gap, the aim of this case report is to detail a fully digital, CBCT‐guided workflow—from diagnosis and planning to guided mini‐implant placement—and comprehensively quantify the 3D skeletal, dentoalveolar, and facial soft‐tissue outcomes of a completely tooth‐free C‐expander inserted laterally into the palatal alveolar process in an adolescent patient.

## 2. Case Report

### 2.1. Diagnosis and Etiology

A 15‐year‐old female patient presented for orthodontic treatment, stating her desire to “improve her smile.” Her medical history included turbinate hypertrophy and allergic rhinitis. Extraoral clinical examination revealed mandibular deviation to the left, and when smiling, pronounced negative spaces with adequate vertical exposure of the incisors. Intraorally, the maxilla was observed to be triangular in shape, deep, with anterior crowding, a Class I molar and canine relationship on the left, a Class III molar and canine relationship on the right, a 1‐mm overjet, a 5% overbite, a posterior crossbite on the premolars, an accentuated curve of Wilson due to the buccal inclination of the upper molars and the lingual inclination of the lower molars, a 1‐mm right midline deviation, and a thin gingival phenotype (Figure 1).

**Figure 1:**
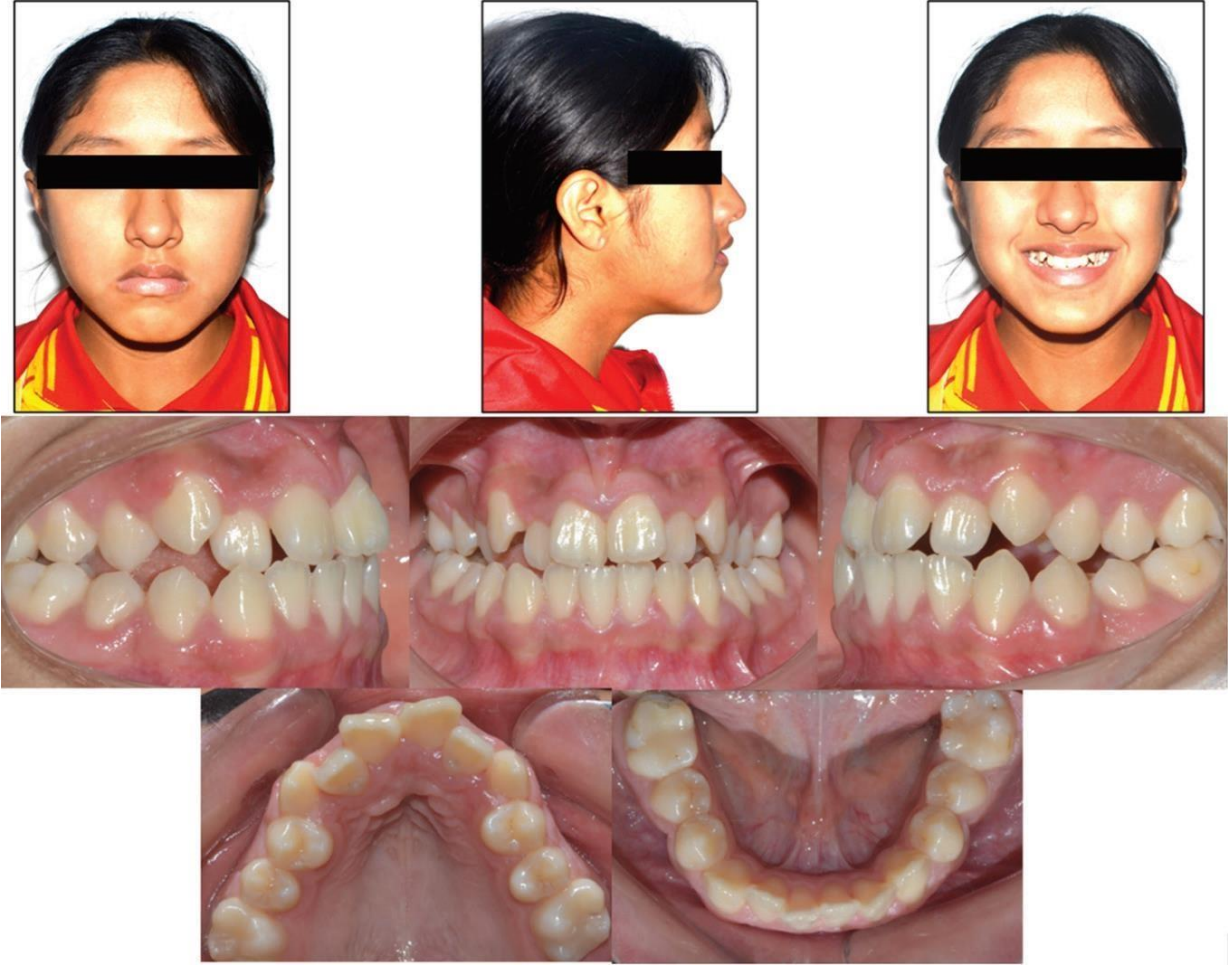
Pretreatment facial and intraoral photographs. The patient presents with a narrow smile, a triangular palate, and lingual inclination of the mandibular posterior teeth.

The imaging analysis included a panoramic radiograph, an intraoral scan, and a CBCT, skeletal, dentoalveolar, and soft‐tissue parameters were evaluated (Table 1).

**Table 1:** Description of evaluated parameters.

Evaluation	Parameter	Description
Skeletal	Maxillary transverse distance	Distance measured in the axial CBCT view between the centers of resistance of the maxillary first molars (center of resistance located at the level of the furcation, triangulated in the axial, sagittal, and coronal views).
	Anterior intersutural distance	Distance measured in the axial CBCT view between the right and left halves of the anterior nasal spine during maxillary expansion.
	Posterior intersutural distance	Distance measured in the axial CBCT view between the right and left halves of the posterior nasal spine during maxillary expansion.
	Mandibular transverse distance	Distance measured in the axial CBCT view between the centers of resistance of the mandibular first molars (center of resistance located at the level of the furcation, triangulated in the axial, sagittal, and coronal views).

Dentoalveolar	Maxillary intermolar distance	Distance measured on the intraoral scan from the mesiopalatal cusp of the right maxillary first molar to the mesiopalatal cusp of the left maxillary first molar.
	Angulation between upper molars	Angle measured in the frontal CBCT view formed between the long axes of the maxillary first molars.
	Mandibular intermolar distance	Distance measured on the intraoral scan from the central fossa of the right mandibular first molar to the central fossa of the left mandibular first molar.
	Angulation between lower molars	Angle measured in the frontal CBCT view formed between the long axes of the mandibular first molars.
	Overjet	Horizontal distance measured on the intraoral scan from the incisal edge of the most prominent maxillary central incisor to the labial surface of the corresponding mandibular incisor.
	Overbite	Vertical distance measured on the intraoral scan from the incisal edge of the mandibular incisor to the vertical projection of the incisal edge of the maxillary incisor.

Soft tissue	Intercanthal distance of the eye	Distance measured on the CBCT facial reconstruction between the inner canthus of the right eye and the inner canthus of the left eye.
	Nasal interalar width	Distance measured in the axial CBCT view between the outermost points of the right and left nasal alar curvatures.
	Nasal base width	Distance measured in the axial CBCT view between the outermost points of the right and left nasal bases.

The patient CBCT scan was evaluated using Xelis Dental Viewer 1.0 software (Infinitt Healthcare, Seoul, South Korea). The images were standardized by orienting them along three reference planes: the palatal plane in the sagittal view, the anterior nasal spine–odontoid process axis in the axial view, and the inferior orbital rim in the frontal view. For facial reconstruction, the frontal view parameters were adjusted using volume rendering (VR) with a 30‐mm slice thickness and the “NewTom skin_01” opacity preset.

To determine the transverse discrepancy of the maxilla in the axial view, the Yonsei analysis was performed [21], obtaining a maxillomandibular difference of −9.1 mm, which corresponds to a net transverse maxillary discrepancy of −6.8 mm. In the coronal view, the molar inclinations were measured, finding a divergence of the upper molars at 24° and a convergence of the lower molars at 31° (Figure 2A). The sutural maturity stage was verified according to Angelieri et al. [11], and the patient was found to be in Stage C (Figure 2B). In addition, the height of the palate was measured, revealing a thin palate, measuring the midsagittal area of the maxilla at 2.28 mm anteriorly and 1.36 mm posteriorly (Figure 2C). In a coronal view, we measured the parasutural areas at the level of the first molars, estimating where the screws of a prefabricated MARPE expander would go, finding a thickness of 0.86 mm on the right and 1.29 mm on the left, in addition to hypertrophy of the nasal turbinate on the right side that would prevent a greater insertion of future miniscrews. A good amount of bone was evaluated at the lateral level of the palate between the buccal cortical bone and the maxillary sinus cortical bone (Figure 2D). Facial reconstruction was performed, and clinical distances were measured at the frontal, intercanthal, internal level of the eyes, nasal interalar, and the distance of the nasal base (Figure 2E). Additionally, an intraoral scan of the maxilla was requested (Figure 2F).

**Figure 2:**
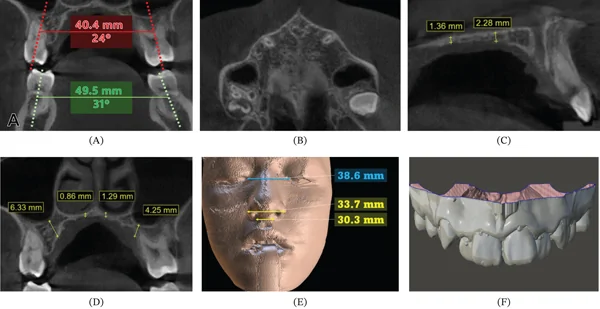
Imaging analysis: (A) CBCT coronal section showing molar distance and inclination; (B) CBCT axial view; (C) CBCT midsagittal section; (D) CBCT coronal section showing palatal thickness; (E) 3D facial reconstruction; (F) maxillary intraoral scan.

After the analyses, we concluded that the definitive diagnosis was a patient with Class I malocclusion due to an alveolodental discrepancy with transverse maxillary deficiency of 6.8 mm.

### 2.2. Treatment Objective

The primary objective was to correct the transverse maxillary deficiency through skeletal expansion, minimizing undesirable dentoalveolar effects. Secondary objectives included improving smile width, optimizing the maxillary arch form, and creating favorable conditions for subsequent comprehensive orthodontic treatment.

### 2.3. Treatment Alternatives

Due to the patient′s age, advanced sutural maturation, scant palatal parasutural bone thickness, and thin periodontal phenotype, maxillary expansion with skeletal anchorage was chosen. The prefabricated MARPE appliances available on the market are anchored paramedially. In contrast, our patient exhibits very thin bone (averaging 1 mm) and turbinate hypertrophy, which would limit bicorticality of the nasal floor. Therefore, an alternative skeletal expander was sought, one with screws inserted laterally, taking advantage of better bone thickness and bicorticality with the maxillary sinus without damaging adjacent teeth (Figure 2D). The C‐expander appliance was selected, which, in addition to allowing lateral screw insertion, is a customized alternative that does not depend on dental anchorage [19, 20].

### 2.4. Treatment Progress

We started treatment by designing the C‐expander on CBCT. For this, we used the BlueSky Plan software (Version 4.9.1, Bluesky Bio, Libertyville, Illinois). We opened the DICOM file in the software and customized the viewing field to only the maxilla (from above the base of the nasal fossae to below the occlusal surface of the molars). We then visualized the different views. We continued with the segmentation of the maxillary model and the separated teeth (Figure 3A). In the 3D reconstruction window, we hid the surface corresponding to the maxillary model and changed the 3D reconstruction configuration to MIP, revealing the maxillary teeth against the CBCT background (Figure 3B). To begin the virtual installation of the miniscrew, we select the add implant button and include from the catalog the one that best suits our requirements. In the tomography, we place the miniscrews taking into account the safe palatal insertion zones [22], safe vertical distance to the bone crest in posterosuperior teeth [23], and the interradicular distance [24] (Figure 3B). We must also verify that the bicorticality required for better stability and force distribution is generated [25] (Figure 3C). We perform the same procedure for four miniscrews, also evaluating that there is not excessive proximity between the screw heads that could pose difficulty later when generating the guide tube (Figure 3B–D). To generate a guide for inserting the miniscrews, the STL file of the maxillary scan is imported (Figure 3E,F), assigning a guide tube to the miniscrews that has a height and displacement of 8 mm, selecting in the guide panel to draw the curve that will follow the desired shape and creating the miniscrew insertion guide (Figure 3G), which we export to an STL file and send to its 3D printing with transparent resin (Figure 3H).

**Figure 3:**
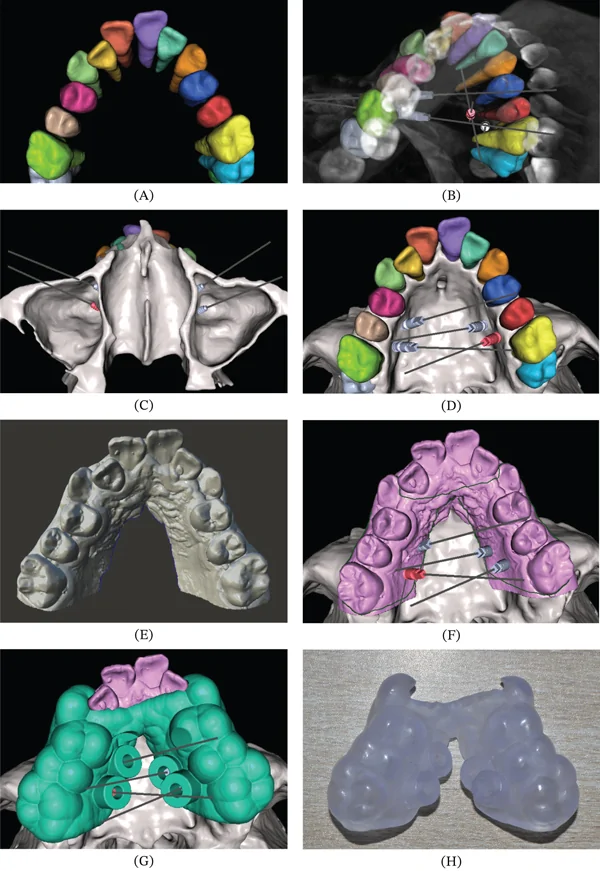
Virtual positioning of miniscrews and fabrication of the surgical guide. (A) Segmentation of maxillary teeth, (B) virtual placement of miniscrews, (C) evaluation of bicorticality in the maxillary sinuses, (D) segmented maxilla with miniscrews, (E) maxillary intraoral scan, (F) intraoral scan superimposed on CBCT, (G) surgical guide design, and (H) printed surgical guide.

To quantify the dimensions of miniscrews in the CBCT, a frontal image was reconstructed by measuring the transmucosal height and thread dimensions up to 1 mm beyond the bicortical space. We obtained anterior miniscrew dimensions of 14‐mm thread, posterior dimensions of 12 mm, and a transmucosal dimension of 4 mm for all, with a 2‐mm diameter (Figure 4A).

**Figure 4:**
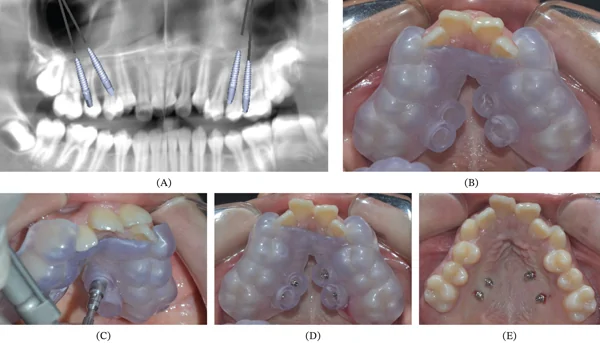
Miniscrew installation workflow. (A) Evaluation of miniscrew height on a coronal CBCT view, (B) surgical guide try‐in, (C) miniscrew insertion, (D) miniscrews in place, and (E) completed installation.

With the prepared and autoclaved guide and the miniscrew dimensions obtained, the clinical procedure begins with asepsis using a chlorhexidine rinse for 1 min. The guide′s stability is tested (Figure 4B), and the correct fit of the miniscrew insertion driver is verified. Infiltrative anesthesia is applied through the guide holes, and the steel miniscrews (Morelli, Sorocaba, São Paulo, Brazil) are inserted, with the driver attached to the motor set at 30 rpm and 35 Ncm of torque until it is close to contacting the mucosa (Figure 4C). This is repeated for all screws (Figure 4D). The guide is then removed, and the insertion is completed manually with the torque wrench until a slight surface of the transmucosal profile is exposed (Figure 4E).

Once the miniscrews were installed, we took an impression of the upper jaw (Figure 5A). The screw areas were relieved with wax, the model was isolated, and the C‐expander appliance was fabricated using self‐curing acrylic and a 7.5‐mm maxillary expansion screw (Dentaurum, Ispringen, Germany), positioned at the level of the first molars and as deep as possible in the palate (Figure 5B). The appliance was tried in, and the holes corresponding to the miniscrews were sealed one by one with flowable resin (Figure 5C,D).

**Figure 5:**
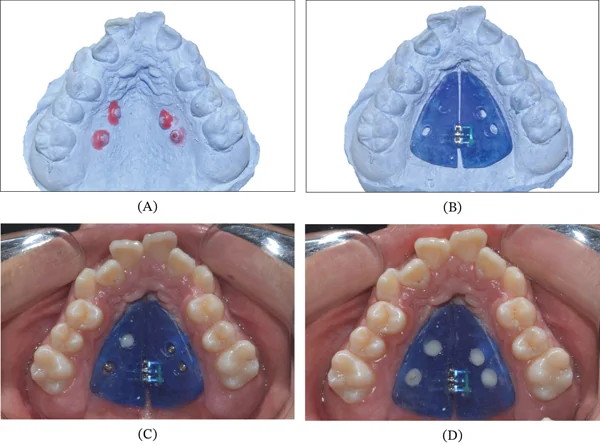
C‐expander placement. (A) Model with miniscrews, (B) C‐expander assembly, (C) C‐expander try‐in, and (D) C‐expander resin sealing.

After installation, the patient and her mother were informed of the necessary hygiene protocol, including gentle brushing and chlorhexidine mouthwash, to prevent insertion failure due to inflammation. She was scheduled for a follow‐up appointment in 1 week to begin activation. After 1 week, when the patient returns, she is instructed to activate 1/4 turn of the expander screw in the mornings and to come for weekly check‐ups.

Weekly checkups were performed; after 14 days, the midincisal diastema was evident; after 28 days of continuous activation, with 7 mm of screw opening, contact was achieved between the palatal cusps of the upper molars and the buccal cusps of the lower molars (Figure 6A,B,C,E). The patient also reported improved breathing and a slight widening of the nasal alae. Given the clinical signs of successful expansion and in order to complete the active phase, the expansion screw was sealed with flowable resin.

**Figure 6:**
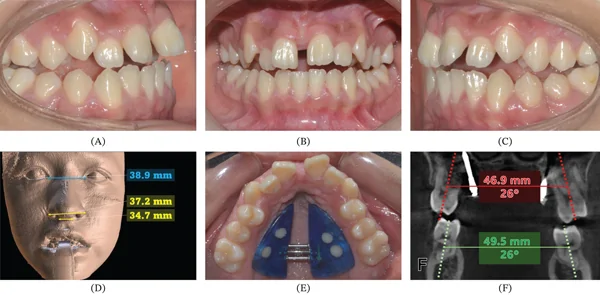
Treatment outcomes with the C‐expander. (A) Right lateral intraoral view, (B) frontal intraoral view, (C) left lateral intraoral view, (D) soft‐tissue evaluation on 3D CBCT reconstruction, (E) maxillary occlusal intraoral view, and (F) molar expansion and inclination evaluation on CBCT.

### 2.5. Results

Postexpansion clinical and CBCT evaluations demonstrated complete resolution of the maxillary transverse deficiency, accompanied by limited dentoalveolar and soft tissue effects. This follow‐up tomographic analysis used identical reference plans and parameters to those established during the baseline assessment.

The treatment outcomes were categorized into skeletal, dentoalveolar, vertical/sagittal, and soft‐tissue parameters:

— Skeletal changes: Postexpansion CBCT verified the bicortical engagement of all four laterally inserted miniscrews within the palatal alveolar process (Figure 7A). Coronal CBCT sections confirmed a basal maxillary skeletal width expansion of +6.5 mm, increasing from 40.4‐mm pretreatment to 46.9‐mm postexpansion (Table 2 and Figure 6F). Axial CBCT view demonstrated an approximately parallel opening pattern of the midpalatal suture along its anteroposterior extent, reaching 6.6‐mm anteriorly and 6.5‐mm posteriorly postexpansion. Although baseline anterior and posterior intersutural distances were reported as 0 mm, this pretreatment value denotes the absence of sutural separation rather than a literal anatomical space of zero width (Table 2 and Figure 7B). In contrast, 3D frontal reconstructions and coronal views revealed a suture opening pattern that was less parallel than the one observed in the axial view (Figure 7C).
— Dentoalveolar changes: Total maxillary intermolar width (measured at the mesiopalatal cusps) increased by +7.1 mm, from 45.2 to 52.3 mm (Table 2). Upper molar buccal inclination increased by only +2° (from 24° to 26°), confirming limited dental tipping (Table 2 and Figure 6F). Spontaneous mandibular dentoalveolar compensation was observed, with lower molars uprighting by +5° (from −31° to −26°) (Table 2 and Figure 6F). Maxillary intercanine distance increased by +4.3 mm (from 33.0 to 37.3 mm) (Table 2). Clinical examination at 28 days of activation revealed a prominent midincisal diastema corresponding to the 7.0‐mm expansion screw opening (Figure 6A–C,E).
— Vertical and sagittal occlusal changes: Overjet increased by +1.0 mm (from 0.5 to 1.5 mm) (Table 2). Overbite decreased by −15% (shifting from +5% pretreatment to −10% postexpansion), resulting in a temporary anterior open bite/edge‐to‐edge relationship caused by initial palatal cusp premature contacts during crossbite uncrossing and secondary clockwise mandibular rotation (Table 2 and Figure 6A–C).
— Soft‐tissue changes: Frontal 3D CBCT reconstructions showed immediate postexpansion increases in nasal base width (+4.4 mm, from 30.3 to 34.7 mm) and interalar width (+3.5 mm, from 33.7 to 37.2 mm), with limited change in intercanthal distance (+0.3 mm, from 38.6 to 38.9 mm) (Table 2 and Figure 6D).

**Table 2:** Parameters evaluated pretreatment and immediately postexpansion.

Parameter category	Measurement	Pretreatment	Immediate postexpansion	Change
Skeletal parameters	Maxillary transverse distance	40.4 mm	46.9 mm	+6.5 mm
	Anterior intersutural distance	0 mm	6.6 mm	+6.6 mm
	Posterior intersutural distance	0 mm	6.5 mm	+6.5 mm
	Mandibular transverse distance	49.5 mm	49.5 mm	0 mm

Dentoalveolar parameters	Maxillary intermolar distance (mesiopalatal cusp)	45.2 mm	52.3 mm	+7.1 mm
	Angulation between upper molars	+24°	+26°	+2°
	Mandibular intermolar distance (central fossa)	51.5 mm	52.5 mm	+1 mm
	Angulation between lower molars	−31°	−26°	+5°
	Overjet	0.5 mm	1.5 mm	+1 mm
	Overbite	5%	−10%	−15%

Soft tissue parameters	Intercanthal distance of the eye	38.6 mm	38.9 mm	+0.3 mm
	Nasal interalar width	33.7 mm	37.2 mm	+3.5 mm
	Nasal base width	30.3 mm	34.7 mm	+4.4 mm

**Figure 7:**
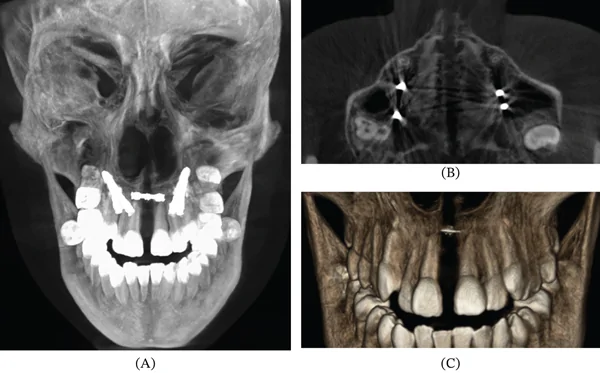
Postexpansion CBCT images: (A) 3D skull reconstruction, (B) coronal view, and (C) axial view.

One month after passive sealing of the expansion screw, spontaneous closure of the median interincisal diastema was observed. The C‐expander was maintained in place as a passive transverse retainer to support subsequent comprehensive fixed orthodontic therapy (Figure 8).

**Figure 8:**
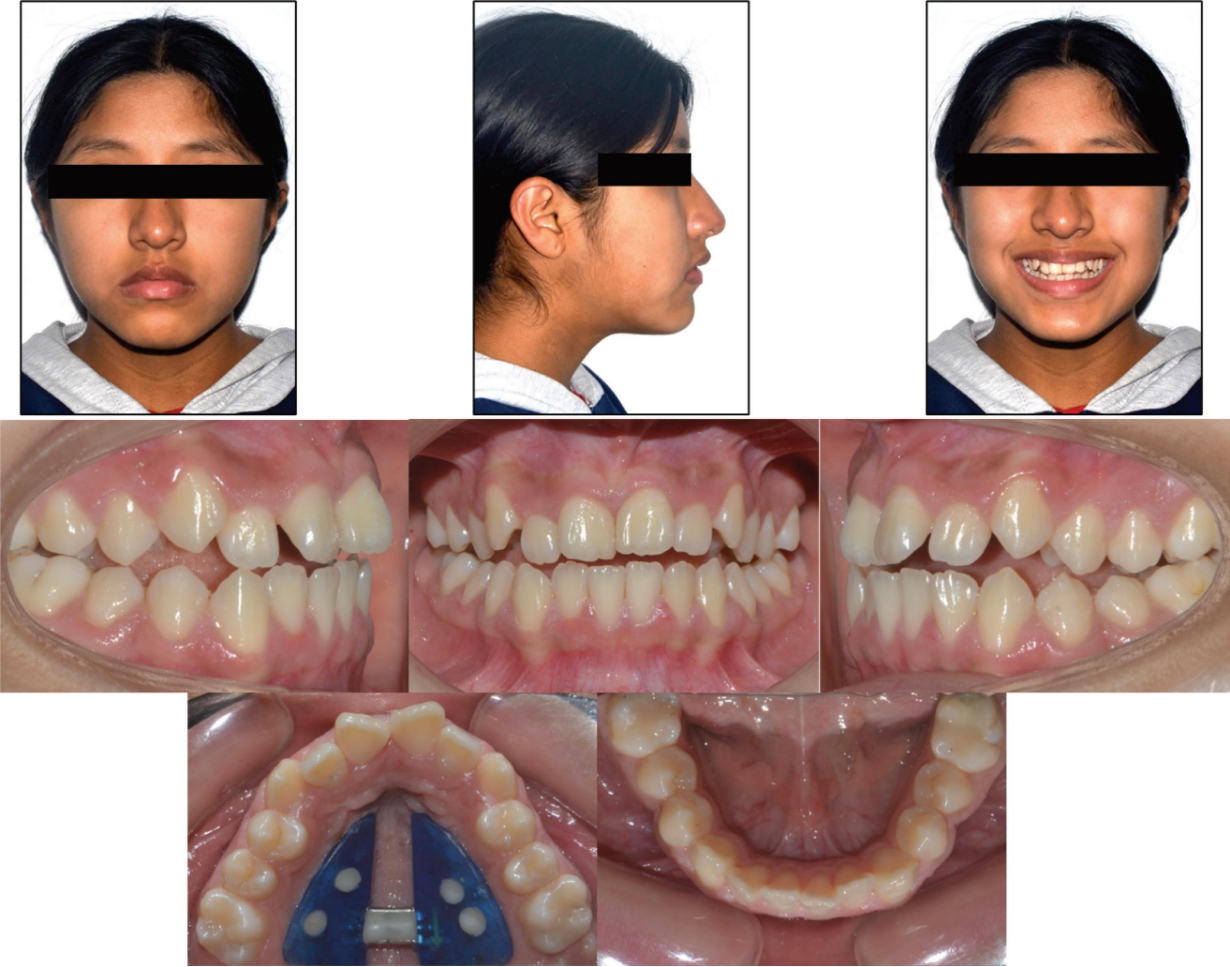
Facial and intraoral photographs at 1 month post‐expansion. The patient presents with a wider smile, an oval palate, and improved maxillomandibular correspondence.

## 3. Discussion

The objective of this case report was to describe the treatment of a patient with the C‐expander appliance, including her diagnosis, treatment planning procedure, fabrication of a secure guide for miniscrew placement, and clinical evolution.

Due to the C‐stage of sutural maturation and the resistance of the midpalatal suture—which increases the risk of adverse effects on tooth anchorage—miniscrew‐assisted expansion was planned from the outset. However, after evaluating the reduced parasutural thickness of the palate, which could limit miniscrew stability [18], and the delicate periodontal phenotype of the molars, which increases the likelihood of undesirable side effects in tooth‐supported MARPE appliances [17], prefabricated hybrid designs were ruled out. Consequently, the C‐expander was selected as a customized, implant‐supported alternative without tooth anchorage. This device utilizes four miniscrews (though reports indicate it can accommodate up to six) inserted laterally to the interradicular alveolar process, establishing a favorable force vector that enhances stability [19, 20]. Furthermore, although the C‐expander is a cost‐effective device compatible with any miniscrew system, its primary disadvantage is the increased difficulty and time required for removal, as the acrylic must be meticulously ground down to access and extract each screw [18–20].

Extraorally, an improvement in the smile was observed, with better width and a decrease in negative spaces, which is evidence of the solution of the transverse discrepancy (Figure 8) [2]; the width of the smile and spontaneous improvement of the crowding was noticed by the patient as part of the solution to her reason for consultation, being satisfied with this first phase. Evaluating the impact at the facial level, assuming that changes in the skeletal structures are reflected in the soft tissues, with great importance in these times of high aesthetic demand from orthodontic patients [26], the images of the 3D reconstructions of CBCT in the frontal plane were compared. As this is a reliable and precise method according to background to determine facial changes [27], we found an increase in the distances at the level of the nasal base by 4.4 mm, nasal interalar by 3.5 mm, and internal intercanthal of the eye by 0.3 mm; these values found can be attributed to the impact of the skeletal change in the frontal plane, in a larger triangular pattern at the level of the nasal base, (Figure 7A,C) with the highest values being those that are closer to the expansion zone. Although there is a clinically relevant change [28], in the long term, this tissue change would largely resolve without aesthetic repercussions [29].

The main objective of the C‐expander is orthopedic expansion with MARPE therapy. In the axial tomographic view (Figure 7B), a nearly parallel opening pattern is observed throughout the entire length of the presented case. This pattern differs slightly in the coronal view, where the opening appears more pyramidal (Figure 7A,C). Background information explains that the C‐expander induces alveolar bone flexion [17, 25], as seen in the coronal view, in addition to the increased buccal displacement of the second molar postexpansion.

The posttreatment maxillomandibular difference was quantified using the Yonsei index, and no discrepancy was found. The success in this clinical case is attributed to the skeletal support and bicorticality achieved in the maxillary sinus cortical bone (Figures 3C and 7A) [25].

Dentally, an improvement in intermolar distance of +7.1 mm was obtained, this as a result of maxillary expansion and vestibular inclination both contributing to a greater transverse distance; regarding sagittal and vertical dentoalveolar outcomes, an overjet increase of 1 mm alongside a 15% reduction in overbite (from +5% to −10%) was observed, resulting in a tendency toward an anterior open bite, this vertical shift occurred during the uncrossing of the posterior crossbite, as premature contact between the palatal cusps of the upper molars and the buccal cusps of the lower molars induced a slight clockwise mandibular rotation and a secondary increase in lower anterior facial height [6]. Although spontaneous uprighting of the lower molars (+5°) occurred as a compensatory response [30], complete occlusal settling requires second‐stage fixed orthodontic appliances.

It is recommended to keep the expansion appliance passively sealed throughout the fixed orthodontic treatment, monitoring oral hygiene and palatal mucosal inflammation. During this phase, complete mineralization of the midpalatal suture is theoretically expected based on existing literature; reports such as Naveda et al. [31] indicate that in young adult patients undergoing expansion with skeletal anchorage, only 50% of the suture ossifies within 16 months—highlighting that this represents a theoretical expectation rather than a directly tracked or long‐term verified outcome in this specific patient. Furthermore, the C‐expander does not interfere with the dentition, allowing for the simultaneous use of fixed appliances.

At the end of treatment, or if inflammatory complications arise around the appliance, it will be removed. This is usually more complex than with other expansion appliances because it requires anesthesia, grinding down the acrylic around the miniscrews, and finally extracting each screw [20].

In addition to the above, although this report is successful, it should not be generalized because it is based on a single case report of a patient at suture maturity stage C who does not present significant ossification in the palatal medullary suture. However, it could be a starting point for establishing a more effective protocol for miniscrew installation and for expanding its use, recognizing its advantages and disadvantages. Furthermore, it is important in future studies to evaluate aspects of this appliance such as its stability, effects according to vertical patterns, and in larger samples of patients with diverse stages of suture maturity.

## 4. Conclusions

In this patient, C‐expander therapy for maxillary transverse deficiency achieved skeletal expansion with limited dental and facial effects. Although these findings cannot be generalized, they suggest that the C‐expander represents a promising alternative to hybrid MARPE appliances in carefully selected cases, provided that meticulous treatment planning with customized surgical guides is used for the placement of the mini‐implants.

## Author Contributions

Enrique Miranda‐Astocondor: conceptualization, investigation, methodology, data collection, writing original draft, and review. Alberto Rafael Ríos‐Quimper: writing original draft, methodology, and review. Marjorie Regina Eguren‐Langer: writing original draft and review.

## Funding

No funding was received for this manuscript.

## Consent

The authors confirm that the patient′s informed consent was obtained from the patient and legal guardians for the inclusion of images and other clinical data in the publication. The patient is aware that every effort will be made to protect her identity, although absolute anonymity cannot be guaranteed.

## Conflicts of Interest

The authors declare no conflicts of interest.

## Supporting information


**Supporting Information** Additional supporting information can be found online in the Supporting Information section.

## Data Availability

The data that support the findings of this study are available from the corresponding author upon reasonable request.
